# Interface-Induced WSe_2_ In-plane Homojunction for High-Performance Photodetection

**DOI:** 10.1186/s11671-020-03342-9

**Published:** 2020-05-14

**Authors:** Jiawei Chi, Nan Guo, Yue Sun, Guohua Li, Lin Xiao

**Affiliations:** 1 Qian Xuesen Laboratory of Space Technology, China Academy of Space Technology, Beijing, 100094 China; 2 Department of Materials Science and Engineering, School of Mechanical Electronic & Information Engineering, China University of Mining & Technology, Beijing, 100083 China

**Keywords:** Transition metal dichalcogenides, In-plane homojunction, Photodetection, Interface gate

## Abstract

2D transition metal dichalcogenides (TMDCs) have been extensively attractive for nano-electronics and nano-optoelectronics due to their unique properties. Especially, WSe_2_, having bipolar carrier transport ability and sizable bandgap, is a promising candidate for future photodetectors. Here, we report an in-plane WSe_2_ homojunction formed by the interface gate of the substrate. In this architecture, an insulated h-BN flake was used to make only part of WSe_2_ flake contact substrate directly. Finally, the structures of WSe_2_/substrate and WSe_2_/h-BN/substrate construct an in-plane homojunction. Interestingly, the device can operate in both photovoltaic and photoconductive modes at different biases. As a result, a responsivity of 1.07 A W^−1^ with a superior detectivity of over 10^12^ jones and a fast response time of 106 μs are obtained simultaneously. Compared with previously reported methods adopted by chemical doping or electrostatic gating with extra bias voltages, our design provides a more facile and efficient way for the development of high-performance WSe_2_-based photodetectors.

## Introduction

In recent decade, 2D transition metal dichalcogenides (TMDCs) have drawn great attention owing to their particular properties. High in-plane mobility, tunable bandgap, mechanical flexibility, strong light-matter interaction, and easy processing make them very competitive for future nano-optoelectronics devices [1–20]. Especially, tungsten diselenide (WSe_2_), a bipolar semiconductor with facile carrier-type manipulation, allows remarkably potential applications in the junction-based photodetectors [21–28]. So far, the main strategies of constructing junction solely in WSe_2_ include chemical doping and electrostatic gating. For example, recently, an intramolecular WSe_2_ p-n junction was reported [26]. The n region and p region within WSe_2_ were formed by polyethyleneimine chemical doping and back gate control, respectively. The p-n junction presented a responsivity of 80 mA W^−1^ and 200 μs response time. Sun et al. doped WSe_2_ by using cetyltrimethyl ammonium bromide to form intramolecular p-n junction, in which the responsivity and the response time are 30 A W^−1^ and ~ 7 ms, respectively [27]. Baugher et al. demonstrate a lateral WSe_2_ p-n junction achieved by electrostatic gating through applying two gate biases with opposite polarity. The responsivity of 210 mA W^−1^ has been obtained [28]. However, due to the inevitable chemical impurities and the necessary multiple bias settings, these methods make the fabrication and application of junction-based devices complex and difficult. Assembling various 2D materials to build vertical van der Waals heterostructures like WSe_2_/MoS_2_ junction [29] has become popular for the development of novel photodetectors. But, in this configuration, the process of carrier transport between different layered materials suffers from the interface defects, which restricts the device response speed. For the Schottky junction formed between metals and 2D materials, the Schottky barrier height is usually determined by Fermi-level pinning, which is uncontrollable and has a great impact on the responsivity of the devices. Additionally, the reported works cannot seem to possess both high responsivity and fast response speed.

Here, we demonstrate a facile and more efficient way to realize an in-plane WSe_2_ homojunction. In the architecture, part of WSe_2_ channel is on the Si/SiO_2_ substrate and the other part is on the h-BN flake. This scheme is common in floating/semi-floating gate memories, in which the h-BN is adopted as gate dielectric layer [30, 31]. The charges stored on one side of h-BN layer can regulate the conductivity of the material on the other side. In our work, however, the h-BN flake as a perfect isolator is used to eliminate the interface gating effect on the WSe_2_ channel. The polarity of WSe_2_, which part is only on the Si/SiO_2_ substrate, can be modulated by interface gate. As a result, the devices operate in photovoltaic (PV) mode well at zero bias. Meanwhile, it exhibits photoconductive (PC) characteristics at high bias. A responsivity of 1.07 A W^−1^ with a superior detectivity of over 10^12^ jones and a fast response time of 106 μs are obtained simultaneously without the intricate device design and the risk of introducing additional chemical impurities.

## Results and Discussion

**Fig. 1:**
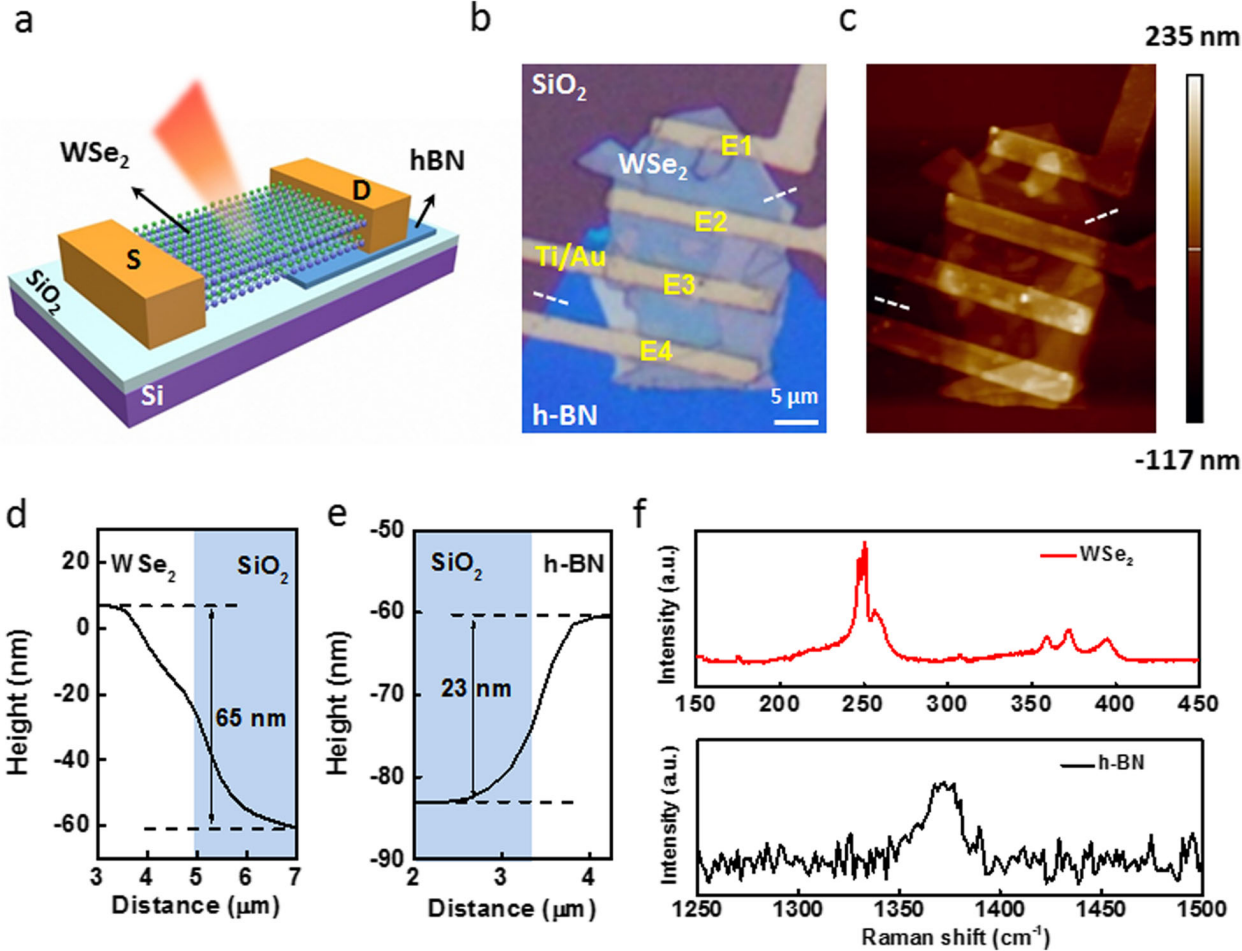
Schematic of an in-plane WSe_2_ homojunction. **a** Structure of the device. **b** Optical image of the device. Part of WSe_2_ contacts h-BN flake while the other part contacts Si/SiO_2_ substrate. **c** AFM image of the device. The white dotted lines indicate the positions where the thickness of h-BN (left) and WSe_2_ (right) are extracted. For the channel between E1 and E2, the average width (length) is ~ 19.15 (~ 6.33) μm. For the channel between E2 and E3, the average width (length) is ~ 23.15 (~ 5) μm. For the channel between E3 and E4, the average width (length) is ~ 22 (~ 5.38) μm. **d**, **e** Height profiles of WSe_2_ and h-BN flakes. **f** Raman spectra of WSe_2_ and h-BN flakes with 532 nm laser excitation

Figure 1a shows a schematic of the in-plane WSe_2_ homojunction. It can be seen that part of WSe_2_ flake is placed on h-BN flake (WSe_2_-h) and the other part contacts the Si/SiO_2_ substrate directly (WSe_2_-S). The function of h-BN is to isolate the interface gate (IG) of the Si/SiO_2_ substrate on the WSe_2_-h. So, the formation of homojunction between WSe_2_-h and WSe_2_-S mainly relies on the IG modulating the polarity of WSe_2_-S. The IG is produced by the trapped charges at the SiO_2_ surface. This will be discussed below in detail. Figure 1b presents the optical picture of the device. Four electrodes (E1-E4, Ti/Au) were prepared by electron-beam lithography, metallization, and the lift-off process. The thickness of materials is characterized by atomic force microscope (AFM) (see Fig. 1c). The height of WSe_2_ (h-BN) flake in direct contact with the Si/SiO_2_ substrate (white dotted lines) was measured as 65 (23) nm (see Fig. 1d, e). It can be seen that there is a slope instead of sharp step in the height profile between the WSe_2_ (h-BN) and the Si/SiO_2_ substrate. This may be due to the residual photoresist at the edge of the material. Figure 1f shows the Raman spectra of WSe_2_ and h-BN flakes. For the WSe_2_, the first order E_2g_ and A_1g_ Raman modes are clearly distinguished ~ 250 cm^−1^, suggesting that the WSe_2_ has a multilayer morphology [32, 33]. For the h-BN, the Raman peak of E_2g_ mode at ~ 1370 cm^−1^ is observed. Due to the large bandgap of h-BN, the Raman signal is weak compared with that in WSe_2_ [34].

**Fig. 2:**
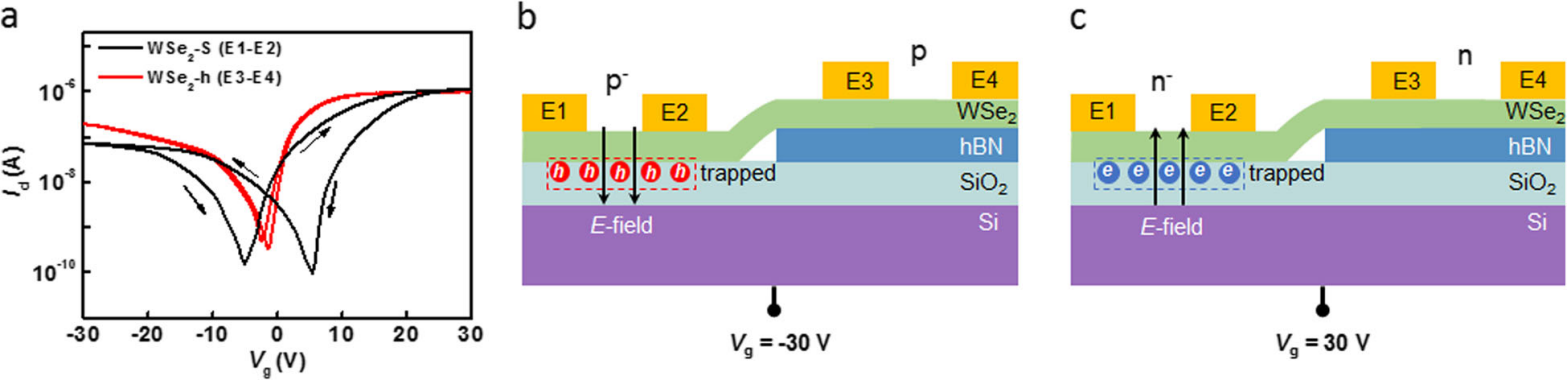
Transfer characteristics. **a***I*_d_-*V*_g_ curves of WSe_2_-S (black line) and WSe_2_-h (red line). The sweep direction of *V*_g_ is indicated by the arrows. **b**, **c** Physical explanation for the hysteresis phenomenon. The arrows indicate the direction of electric field induced by *V*_g_. The red and blue spheres represent holes and electrons, respectively

To explore the effect of substrate on WSe_2_, transfer characteristics of WSe_2_-S and WSe_2_-h were studied separately. As shown in Fig. 2a, both transfer curves exhibit bipolar behavior and an obvious hysteresis can be observed in the curve of WSe_2_-S (black) compared with that of WSe_2_-h (red). The current of WSe_2_-h is higher than that of WSe_2_-S. The steep slope in the curve of WSe_2_-h indicates a relatively large transconductance, which is proportional to carrier mobility. For WSe_2_-S, the hysteresis is attributed to the charge trapping at the SiO_2_ surface [35–38]. When *V*_g_ was swept from − 30 to 0 V, the negative *V*_g_ makes the WSe_2_ populated with holes and drives some holes into the SiO_2_ (see Fig. 2b). The trapped holes in SiO_2_ generate a positive local gate, i.e., IG, to modulate the WSe_2_ conductance in return (weak depletion effect). Therefore, the charge neutrality point of *V*_g_ appears around − 5 V. Similarly, when *V*_g_ was swept from 30 to 0 V, the positive *V*_g_ makes the WSe_2_ populated with electrons and also drives some electrons into the SiO_2_ (see Fig. 2c). The trapped electrons in SiO_2_ generate a negative IG to modulate the WSe_2_ conductance in return (the same weak depletion effect). So, the charge neutrality point of *V*_g_ appears around 5 V. For WSe_2_-h, the h-BN flake inhibits the carrier transfer between WSe_2_ and SiO_2_ under *V*_g_ modulation. This is the reason for the non-obvious hysteresis in the WSe_2_-h curve. Therefore, an in-plane homojunction can be formed simply by taking advantage of the IG.

**Fig. 3:**
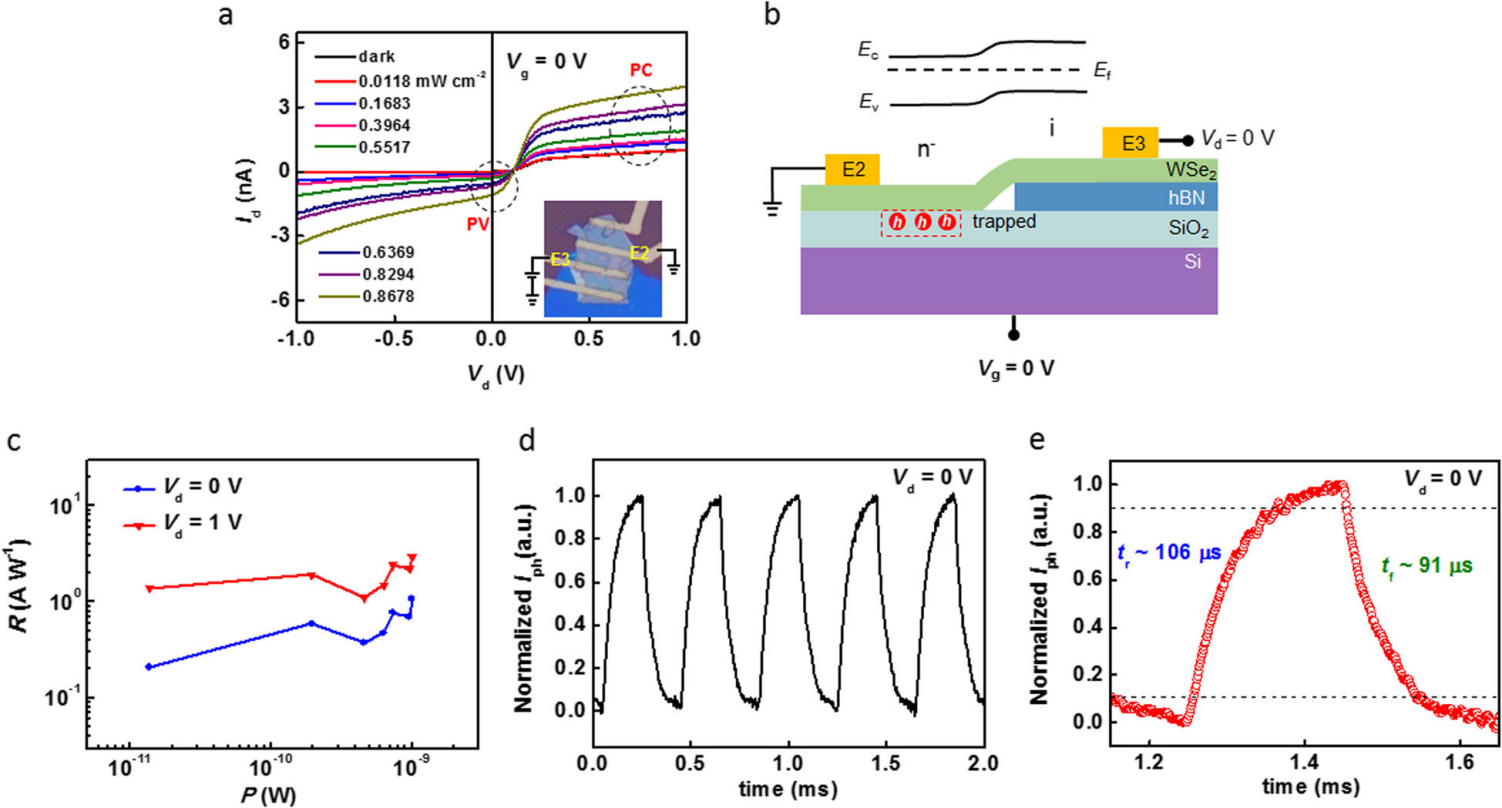
Photoresponse performance of the homojunction acquired between E2 and E3. **a** Drain current as a function of source-drain voltage applied on electrodes E2 and E3 (see the inset) with variable light power intensity (637 nm). **b** Formation mechanism of the homojunction at *V*_g_ = 0 V and *V*_d_ = 0 V. **c** Responsivity as a function of light power. **d**, **e** Temporal response of the device acquired at *V*_d_ = 0 V for 637 nm illumination. An oscilloscope was used to monitor the time dependence of the current

Figure 3a shows the *I*_d_-*V*_d_ curves of the device under dark and light conditions at *V*_g_ = 0 V. The source-drain voltage is applied on electrodes E2 and E3 (see the inset). It can be seen that the short-circuit currents (at *V*_d_ = 0 V) increase with the incident power, indicating a PV effect. Interestingly, the curves also present PC characteristics at *V*_d_ = ± 1 V. For the former, the photocurrents are attributed to the homojunction. As shown in Fig. 3b, although *V*_d_ and *V*_g_ were set at 0 V, a few already trapped holes in SiO_2_ form a small positive IG to modulate the WSe_2_-S. So, the n^-^-type WSe_2_-S and intrinsic WSe_2_-h (without the effect of IG due to the isolation by h-BN flake) constitute an in-plane homojunction. Under illumination, the photoexcited electron-hole pairs will be separated by built-in field of the homojunction. Although *I*_d_-*V*_d_ curves present PV characteristic well at zero bias, the homojunction did not show a rectifying behavior maybe due to the relatively weak built-in field compared with the externally applied *V*_d_. For the latter, the whole WSe_2_ flake as a photoconductor responds the light signal at high bias. The photoexcited carriers will be driven to the electrodes by *V*_d_. Therefore, the photoresponse in Fig. 3a is the result of synergistic effect of PV and PC modes. The responsivities as a function of the light power for different *V*_d_ are summarized in Fig. 3c, given by *R* = *I*_ph_/*PA*, where *I*_ph_ is the photocurrent, *P* is the power intensity, and *A* is the effective photosensitive area of the detector [39, 40]. During the calculation, the effective photosensitive area, i.e., the WSe_2_ part between E2 and E3, is 115.75 μm^2^. The responsivities of 1.07 A W^−1^ and 2.96 A W^−1^ are obtained for *V*_d_ of 0 V and 1 V, respectively. The specific detectivity (D^*^) as an important parameter determines the capability of a photodetector to response a weak light signal. Assuming that the shot noise from the dark current is the major contribution, D^*^ can be defined as *D*^∗^ = *RA*^1/2^/(2*eI*_dark_)^1/2^, where *R* is the responsivity, *A* is the effective photosensitive area, *e* is the electron charge, and *I*_dark_ is the dark current [41, 42]. Benefitting from the extremely low *I*_dark_, D^*^ of 3.3 × 10^12^ jones (1 jones = 1 cm Hz^1/2^ W^−1^) and 1.78 × 10^11^ jones are achieved for *V*_d_ of 0 V and 1 V, respectively. Moreover, response time as a key figure of merit has been studied. As shown in Fig. 3d, a high and a low current state acquired at *V*_d_ = 0 V have been obtained with the light modulation. The transient photoresponse exhibits highly stable and reproducible characteristics. Figure 3e gives a single modulation cycle of temporal response. The rising time (*t*_r_), defined as the time necessary for the current to increase from 10% *I*_peak_ to 90% *I*_peak_, was found to be ~ 106 μs, and the falling time (*t*_f_), defined analogously, was found to be ~91 μs. Figure S1 shows temporal response of the device acquired at *V*_d_ = 1 V. *t*_r_ and *t*_f_ were found to be ~105 μs and ~ 101 μs, respectively. Table 1 summarizes the reported WSe_2_ homojunction formed by different methods. Obviously, the device in our work has high D^*^, comparable *R*, and relatively fast response speed. Moreover, Figure S2 presents the photoresponse characteristics of the other three devices. Distinct PV and PC currents can be observed at zero and high bias, respectively. The detectivity of all the WSe_2_ homojunctions is higher than 10^12^ jones, and the response time is a little more than 100 μs, proving that our devices can repeat the high-performance photodetection very well.

**Table 1:** Optoelectronic characteristics of WSe_2_ homojunction formed by different methods

WSe_2_ homojunction formed by	Wavelength (nm)	Responsivity (A W^−1^)	Detectivity (jones)	Time	References
h-BN/two gates	532	7 × 10^−4^	-	10 ms	24
SiN/two gates	500–900	0.016	-	-	25
Polyethylene imine chemical doping	520	0.08	10^11^	200 μs	26
Cetyltrimethyl ammonium bromide chemical doping	450	30	10^11^	7.8 ms	27
HfO_2_/two gates	532	0.21	-	-	28
h-BN/interface gate	637	1.07	10^12^	106 μs	This work

**Fig. 4:**
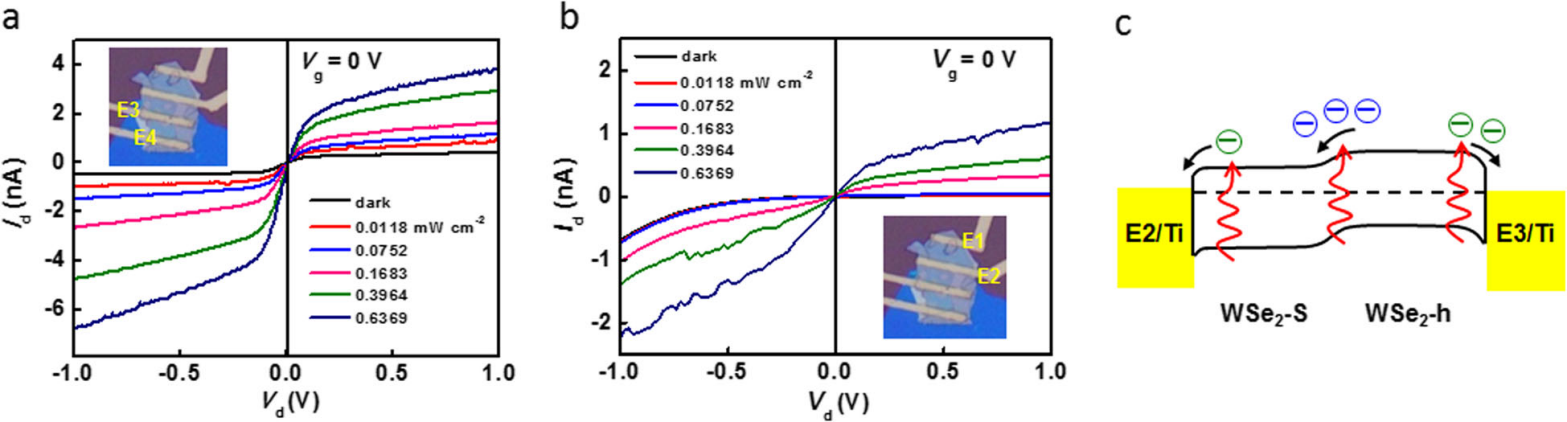
Effect of Schottky junction on photoresponse. **a***I*_d_-*V*_d_ curves of WSe_2_-h with source-drain voltage applied on electrodes E3 and E4 (see the inset) under light illumination (637 nm). **b***I*_d_-*V*_d_ curves of WSe_2_-S with source-drain voltage applied on electrodes E1 and E2 (see the inset) under light illumination (637 nm). **c** Schematic band diagram of the homojunction device with asymmetric Schottky contacts, i.e., E2/WSe_2_-S and E3/WSe_2_-h, at zero bias

Figure 4a and b present the *I*_d_-*V*_d_ characteristics of WSe_2_-h and WSe_2_-S separately. The curves of both WSe_2_-h and WSe_2_-S exhibit PC property, and there is no photocurrent at zero bias. In fact, Ti/WSe_2_/Ti should be supposed to form a metal/semiconductor/metal structure which contains two Schottky junctions with opposite built-in field. So, the *I*_d_-*V*_d_ curves should be cross the zero-point and exhibit PC behavior. In our case, due to the different work functions of WSe_2_-h and WSe_2_-S, there are two asymmetric Schottky contacts, i.e., E2/WSe_2_-S and E3/WSe_2_-h, as shown in Fig. 4c. At zero bias, the direction of net photocurrents originated from the Schottky junctions is opposite to that in the homojunction, and the experiment result shown in Fig. 3a is consistent with the latter. Therefore, the homojunction formed between WSe_2_-h and WSe_2_-S is the reason for the short-circuit photocurrents.

To further demonstrate that the photoresponse at zero bias is attributed to the homojunction, the output properties were investigated through measuring the *I*_d_-*V*_d_ curves of the device with the source-drain voltage applied on electrodes E1 and E4. As shown in Figure S3a, the curves, same as the situation in Fig. 3a, also exhibit the PV and PC characteristics. As discussed above, for the former, the photocurrents are attributed to the built-in field of in-plane homojunction formed between WSe_2_-S and WSe_2_-h. For the latter, the photocurrents are attributed to the collection of photoexcited carriers by the externally applied *V*_d_. The responsivities as a function of the light power for different *V*_d_ are summarized in Figure S3b. The responsivities (detectivities) of 0.51 A W^−1^ (2.21 × 10^12^ jones) and 3.55 A W^−1^ (5.54 × 10^12^ jones) are obtained for *V*_d_ of 0 V and 1 V, respectively. During the calculation, the effective photosensitive area, i.e., the WSe_2_ part between E1 and E4, is 519.4 μm^2^. The response time measured at zero bias is shown in Figure S3c and 3d, in which the rising time is 289 μs and the falling time is 281 μs. For the *V*_d_ of 1 V (Figure S3e and 3f), the rising and falling time are 278 μs and 250 μs, respectively. The response speed is a little slower than that measured between electrodes E2 and E3, because the relatively long conductive channel increases the photocarrier transmission distance and the probability for the interaction between photocarriers and defects.

## Conclusion

In summary, we have demonstrated an in-plane WSe_2_ homojunction by electrically tuning partial WSe_2_ flake through interface gate. Compared with existing approaches like chemical doping and electrostatic gating by taking advantage of two gate biases, this design gives a more facile rout to realize WSe_2_ homojunction. With light illumination, the device produces distinct short-circuit photocurrents with a detectivity of 3.3 × 10^12^ jones. At high bias, the device presents photoconductive characteristic and generates photocurrents with a detectivity of 1.78 × 10^11^ jones. A response time as fast as 106 μs is also obtained simultaneously. Our study provides an efficient and reliable way for the development of high-performance WSe_2_-based photodetectors.

## Methods

Both WSe_2_ and h-BN bulk materials were purchased from Shanghai Onway Technology Co., Ltd. First, the h-BN and WSe_2_ flakes were mechanically exfoliated onto a p^+^-Si/SiO_2_ (300 nm) substrate and a poly-dimethyl siloxane (PDMS) layer, respectively. Then, a micromanipulator was used to put the WSe_2_ flake, which is adhered to PDMS, onto the target h-BN flake through the microscope to locate the position. Part of WSe_2_ flake overlaps the h-BN flake. Finally, the WSe_2_ flake was released from PDMS through heating the substrate. The electrodes (Ti/Au) were prepared by electron-beam lithography, metallization, and the lift-off process. Photoresponse measurements were conducted using Agilent B1500 semiconductor parameter analyzer and laser diode with the wavelength of 637 nm.

## Supplementary information

**Additional file 1: Figure S1.** Temporal response of the device acquired at *V*_d_ = 1 V for 637 nm illumination. **Figure S2.** Photoresponse of the other three devices under 637 nm illumination. **Figure S3** Photoresponse performance of the homojunction acquired between E1 and E4.

## Data Availability

The data that support the findings of this work are available from the corresponding author upon reasonable request.

## Abbreviations

TMDCs: Transition metal dichalcogenides
PV: Photovoltaic
PC: Photoconductive
AFM: Atomic force microscope
IG: Interface gate
PDMS: Poly-dimethyl siloxane
