# Supplementary material for: Interface-Induced WSe2 In-plane Homojunction for High-Performance Photodetection
Source: Nanoscale Res Lett. 2020 May 14;15:111. doi: 10.1186/s11671-020-03342-9 (PMC7225239; doi:10.1186/s11671-020-03342-9)
Supplement: Supplementary file 1 — Additional file 1: Figure S1. Temporal response of the device acquired at Vd = 1 V for 637 nm illumination. Figure S2. Photoresponse of the other three devices under 637 nm illumination. Figure S3 Photoresponse performance of the homojunction acquired between E1 and E4. [file 11671_2020_3342_MOESM1_ESM.docx]

**Supplementary information**

Interface-induced WSe_2_ in-plane homojunction for high-performance photodetection

Jiawei Chi,^1,2^ Nan Guo,^1,*^ Yue Sun,^1^ Guohua Li,^2^ Lin Xiao^1,*^

^1^Qian Xuesen Laboratory of Space Technology, China Academy of Space Technology, Beijing 100094, China

^2^Department of Materials Science and Engineering, School of Mechanical Electronic & Information Engineering, China University of Mining & Technology, Beijing 100083, China

*Corresponding author: guonan@qxslab.cn (N. Guo); xiaolin@qxslab.cn (L. Xiao)

**
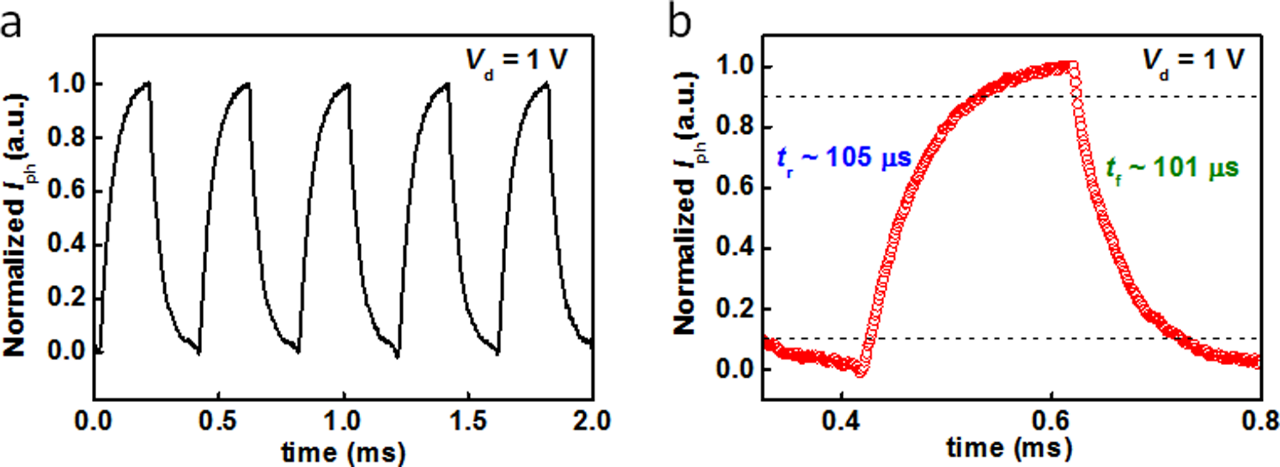
**

**Fig. S1.** Temporal response of the device acquired at *V*_d_ = 1 V for 637 nm illumination.

**
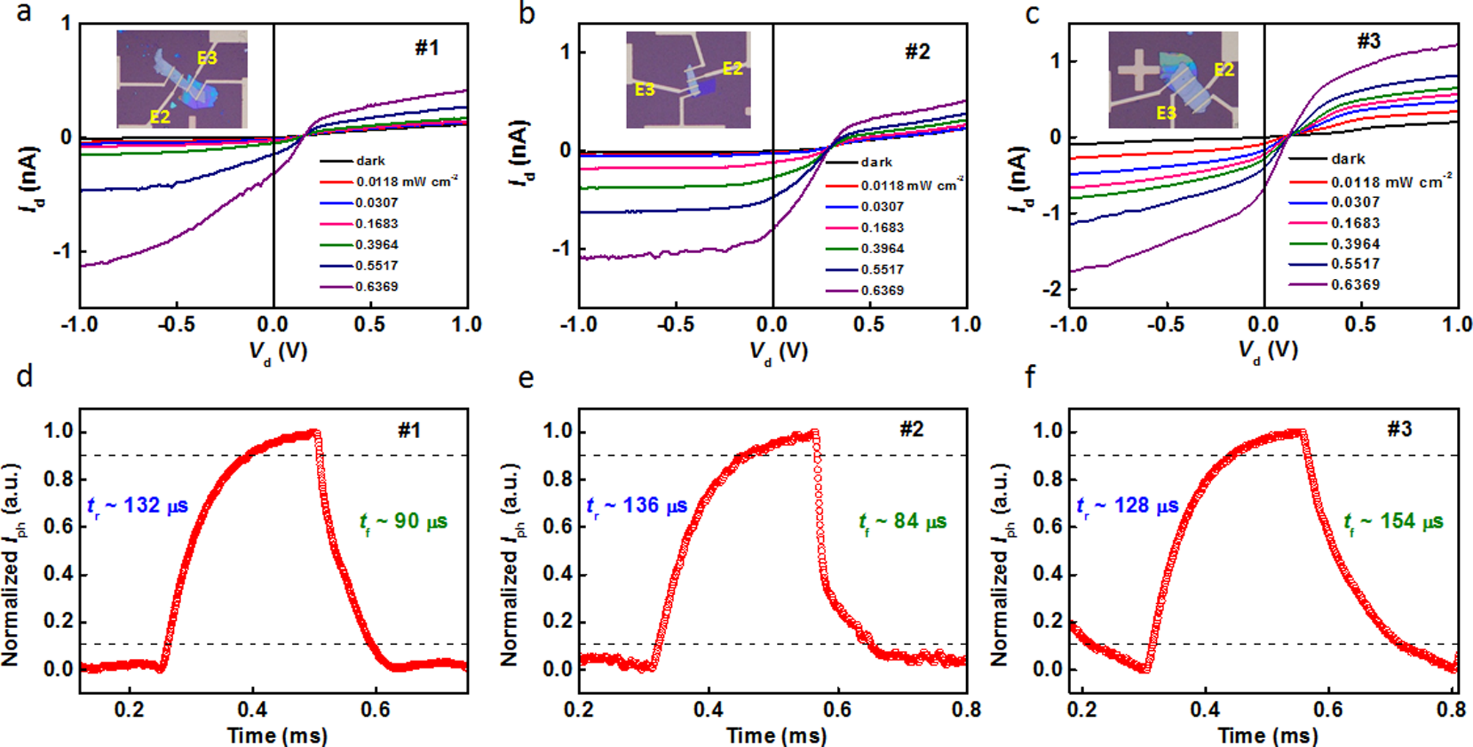
**

**Fig. S2.** Photoresponse of the other three devices under 637 nm illumination. (a,b,c) Drain current as a function of source-drain voltage applied on electrodes E2 and E3 (see the inset). The detectivities of device#1, #2 and #3 acquired at *V*_d_ = 0 V are 3.05×10^12^, 2.6×10^12^ and 5.0×10^12^ jones, respectively. (d,e,f) Temporal response of the devices acquired at *V*_d_ = 0 V.

**
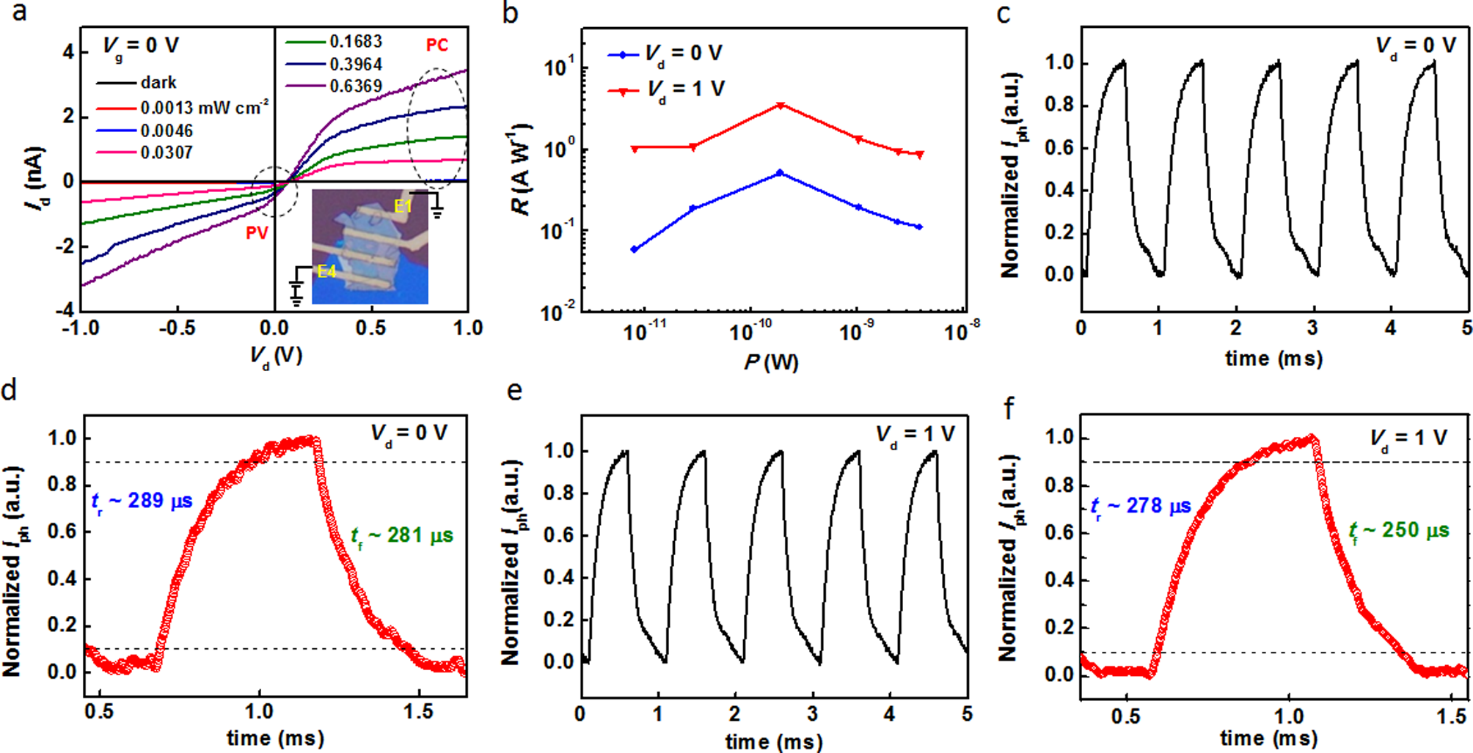
**

**Fig. S3** Photoresponse performance of the homojunction acquired between E1 and E4. (a) Drain current as a function of source-drain voltage applied on electrode E1 and E4 (see the inset) with variable light power intensity (637 nm). (b) Responsivity as a function of light power. (c,d,e,f) Temporal response of the device acquired at *V*_d_ = 0 V and 1 V for 637 nm illumination.
